# Comparison of cancer risk between neutrophilic dermatoses and plaque psoriasis patients: a cancer registry-based study

**DOI:** 10.1007/s00403-023-02703-5

**Published:** 2023-08-15

**Authors:** Alessandro Borghi, Stefano Ferretti, Fabio Falcini, Natale Schettini, Monica Corazza

**Affiliations:** 1 Department of Medical Sciences, Section of Dermatology and Infectious Diseases, University of Ferrara, Via L. Ariosto 35, 44121 Ferrara, Italy; 2 ​Department of Translational Medicine and for Romagna, Emilia-Romagna Cancer Registry, Functional Unit of Ferrara, Local Health Authority Ferrara, University of Ferrara, Ferrara, Italy; 3 Emilia-Romagna Cancer Registry, IRCCS Istituto Romagnolo per lo Studio dei Tumori (IRST) “Dino Amadori”, Local Health Authority, Meldola, Forlì, Italy

**Keywords:** Cancer registry, Cancer risk, Neutrophilic dermatoses, Plaque psoriasis, Population-based cohort study

An increased risk of developing tumors is recognized for patients affected with neutrophilic dermatoses [2–4], while this risk is less explicit for plaque psoriasis [5]. Our objective was to assess and compare, for the first time, the cancer risk between patients with neutrophilic dermatoses and those with plaque psoriasis.

This population-based, retrospective cohort study included all the patients who had received a histological diagnosis of either neutrophilic dermatoses or plaque psoriasis between 1995 and 2015 in the province of Ferrara, northern Italy. It was intended to assess the risk of cancer development based on the Ferrara Cancer Registry dataset.

Patients with prevalent cancers (diagnosed before the diagnosis of plaque psoriasis or neutrophilic dermatoses) and those without a histological confirmation of either plaque psoriasis or neutrophilic dermatosis were excluded. Multiple tumors following the first, both in cohort and in general population subjects, were not considered in risk calculation. The following indices were assessed: (i) standardized incidence ratio (SIR), (ii) individual attributable risk (AR), and (iii) attributable risk among the exposed (ARe).

The study cohort included 278 patients (51.8% males, age at diagnosis ranging from 9 to 93 years, median 54.5), of whom 52 (18.7%) were affected with neutrophilic dermatosis and 226 (81.3%) with plaque psoriasis. The group of neutrophilic dermatoses was represented by Sweet’s syndrome (7 cases), pyoderma gangrenosum (14), hidradenitis suppurativa (4), subcorneal pustulosis (7) and pustular psoriasis (20). We included pustular psoriasis within the cohort of neutrophilic dermatoses due to its immune pathway and inflammatory pattern, which are closer to those observed in these disorders than in plaque psoriasis [1].

During the follow-up (median 10.7 years, total 3005 person*years), 49 patients (17.6%) received a diagnosis of cancer (row incidence rate 1630.6 × 100,000, CI 95% 1206–2156), occurring from 11 months to 20 years from the diagnosis of their skin disease (median 7.6 years) (Table 1).

**Table 1:** Tumors diagnosed in the entire study cohort

Cancer sites	Cases *n*	*%*	Male cases, *n*	Female cases, *n*	Neutrophilic dermatoses Cases, *n*	Plaque psoriasis Cases, *n*
No cancer	229	*82.4*	112	117	41	188
Oral cavity	1	*0.4*	–	1	1	–
Stomach	4	*1.4*	4	–	3	1
Colon	3	*1.1*	1	2	1	2
Liver, biliary tract	3	*1.1*	3	–	1	2
Lung	5	*1.8*	4	1	1	4
Skin (keratinocytic)	7	*2.5*	4	3	1	6
Breast	8	*2.9*	1	7	1	7
Uterus (body)	1	*0.4*	–	1	–	1
Prostate	2	*0.7*	2	–	–	2
Kidney	3	*1.1*	3	–	–	3
Urothelial^a^	6	*2.2*	5	1	1	5
Central nervous system	3	*1.1*	–	3	-	3
Non-Hodgkin lymphomas	1	*0.4*	1	–	–	1
Myelodysplastic syndromes	1	*0.4*	1	–	–	1
Non-specified site	1	*0.4*	1	–	1	–
Total	**49**	***17.6***	**30**	**19**	**11**	**38**

Bold represents the amount of these percentages

Italic represents the percentage of cases described

^a^Renal pelvis, ureter, bladder, urethra (uncertain malignant potential, in situ, invasive)

A first remarkable finding was that the whole cohort experienced a 31% global excess of cancer risk with respect to the age-matched general population living in the same area (Table 2).

**Table 2:** Cancer incidence risk in the cohort patients

	Incident cancers, *n*	Person years^1^	SIR^2^	*CI95%*	AR^3^	Are (%)^4^
Total	49	3005	1.31	*0.97–1.73*	9.2	57
Males	30	1423	**1.48**	***1.0018–2.12***	12.5	59
Females	19	1582	1.10	*0.66–1.72*	60.0	50
Diagnosis^5^ 0–54 years	9	1162	2.14	*0.98–4.07*	5.5	72
55 + years	40	1843	1.21	*0.86–1.64*	3.1	14
Neutrophilic dermatoses	11	571	1.35	*0.68–2.42*	10.7	55
Plaque psoriasis	38	2435	1.30	*0.92–1.79*	9.6	61
Cutaneous carcinoma^6^	7	3005	1.03	*0.42–2.13*	0.7	32
Female breast cancer	7	1582	1.64	*0.66–3.39*	2.5	58

Significant values are reported in bold

Italic is used only to distnguish percentage from absolute values

^1^From Ferrara Unit of Emilia-Romagna Cancer Registry 1995–2017 (70,181 incident cases; 8,113,698 p*y)

^2^Standardized Incidence Ratio (observed/expected*)

^3^Attributable risk × 1000

^4^Attributable risk in exposed (cohort)

^5^Age at histological diagnosis of neutrophilic dermatosis/plaque psoriasis

^6^Keratinocytic forms

With respect to the main study objective, the increased cancer risk among patients with neutrophilic dermatoses (SIR 1.35, CI 95% 0.7–2.4), when compared with the general population, did not differ significantly from that of patients with plaque psoriasis (SIR 1.30, CI 95% 0.9–1.8) both in univariate and multivariate Cox model.

This novel result merits a few considerations. First, it is conceivable that the systemic chronic inflammatory state present in these patients could represent a cofactor of tumor risk in both groups. If chronic inflammation can be considered a plausible background that may lead to carcinogenesis, it should also be emphasized that the inflammatory pathways of neutrophilic dermatoses are considerably different from those of plaque psoriasis. Further studies are expected both to deepen the understanding of the relationship between these inflammatory skin diseases and cancers and to underline any similarities and differences between the two types of inflammatory disorders in the carcinogenic process.

From a practical perspective, a more intense cancer surveillance may be warranted in these patients. This is a somewhat consolidated practice in patients with neutrophilic dermatoses and should ideally be activated for psoriatic patients as well.

## Data Availability

The data that support the findings of this study are available from the corresponding author, N.S., upon reasonable request.

## References

[1] Bachelez H (2018). Pustular psoriasis and related pustular skin diseases. Br J Dermatol.

[2] Marzano AV, Borghi A, Wallach D, Cugno M (2018). A comprehensive review of neutrophilic diseases. Clin Rev Allergy Immunol.

[3] Montagnon CM, Fracica EA, Patel AA, Camilleri MJ, Murad MH, Dingli D, Wetter DA, Tolkachjov SN (2020). Pyoderma gangrenosum in hematologic malignancies: a systematic review. J Am Acad Dermatol.

[4] Nelson CA, Noe MH, McMahon CM, Gowda A, Wu B, Ashchyan HJ, Perl AE, James WD, Micheletti RG, Rosenbach M (2018). Sweet syndrome in patients with and without malignancy: a retrospective analysis of 83 patients from a tertiary academic referral center. J Am Acad Dermatol.

[5] Vaengebjerg S, Skov L, Egeberg A, Loft ND (2020). Prevalence, incidence, and risk of cancer in patients with psoriasis and psoriatic arthritis: a systematic review and meta-analysis. JAMA Dermatol.

